# Complete Genome Sequence of a *Zucchini Yellow Mosaic Virus* Isolated from Pumpkin in Oklahoma

**DOI:** 10.1128/MRA.01583-18

**Published:** 2019-01-10

**Authors:** Vivek Khanal, Akhtar Ali

**Affiliations:** a Department of Biological Science, The University of Tulsa, Tulsa, Oklahoma, USA; Queens College

## Abstract

*Zucchini yellow mosaic virus* (ZYMV) (genus Potyvirus, family *Potyviridae*) was first isolated in Europe during the 1970s and in the United States in 1981. Here, we report the complete genome sequence of ZYMV isolate BL-67, isolated from pumpkin in Oklahoma during the 2016 growing season.

## ANNOUNCEMENT

Cucurbits are commonly grown as cash crops in the United States. The six major cucurbits (cantaloupe, cucumber, honeydews, pumpkin, squash, and watermelon) in the United States generated $1,732 million in 2017, which is 12.5% of the total value of production from the fresh vegetable market (1). *Zucchini yellow mosaic virus* (ZYMV) is a member of the family *Potyviridae* and the genus Potyvirus. (2). During the 1970s, ZYMV was simultaneously described in Italy from zucchini (3) and in France from muskmelon (4). ZYMV has worldwide distribution, mostly in tropical, subtropical, and temperate regions, and members of the family Cucurbitaceae are its primary hosts (5). ZYMV is transmitted in a nonpersistent manner by more than 26 aphid vectors (3, 4, 6) and also by seeds (7, 8).

ZYMV was first described in the United States from squash in 1981 (9). Simmons et al. (10) sequenced the complete genome of a single field isolate of ZYMV from wild gourd (Cucurbita pepo subsp. texana). Subsequently, the same field isolate of ZYMV was transmitted mechanically and by aphid vectors to obtain 24 subisolates of ZYMV, which were also completely sequenced. There are no other complete genome sequences of ZYMV available from any other regions of the United States, despite ZYMV being an emerging viral disease in the country, particularly in the southern states (11–13).

During a survey in the 2016 growing season, leaf samples from plants showing virus-like symptoms were collected from pumpkin fields in Blaine County, Oklahoma. Leaf samples were serologically screened using a dot-immunobinding assay (DIBA) (14) against antisera of ZYMV (AC Diagnostics). Among the several DIBA-positive samples, an isolate named BL-67 was used for molecular characterization of ZYMV. Infected tissues of isolate BL-67 were mechanically inoculated into pumpkin seedlings (15). Two weeks postinoculation, total RNA was extracted from leaves according to the TRI Reagent method (Molecular Research, USA) (15).

Nine pairs of overlapping primers were designed from the complete genome sequences of ZYMV isolates available in GenBank and synthesized commercially (Integrated DNA Technologies, USA). All nine fragments were successfully amplified by reverse transcription-PCR (RT-PCR), confirmed on 1% agarose gels, and cleaned with ExoSap-IT (Affymetrix, USA) (16). Purified PCR products were directly sequenced in both directions using an Applied Biosystems 3130 analyzer at the Department of Biological Science at The University of Tulsa. Nucleotide sequences were visualized using FinchTV version 1.4 (Geospiza Inc., USA) and were aligned using ClustalW (17).

The complete genome of ZYMV was 9,553 nucleotides long, encoding a polyprotein of 3,080 amino acid residues comprising 10 different genes. Nucleotide BLAST searches showed that ZYMV isolate BL-67 showed 83% to 96% nucleotide sequence identity and 86% to 98% amino acid sequence identity with the available ZYMV complete genome sequences in the GenBank database. The highest nucleotide (96%) and amino acid (98%) sequence similarities were observed with a South Korean isolate (GenBank accession no. MH042025). Interestingly, isolate BL-67 showed only 92% nucleotide and 95% amino acid identities with the isolate of ZYMV from the United States (10).

### Data availability.

The complete genome sequence of ZYMV isolate BL-67 was deposited in GenBank under the accession no. MK124612.
